# Ambient Fine Particulate Matter Exposure and Myocardial Ischemia in the Environmental Epidemiology of Arrhythmogenesis in the Women’s Health Initiative (EEAWHI) Study

**DOI:** 10.1289/ehp.0800046

**Published:** 2009-01-23

**Authors:** Zhu-ming Zhang, Eric A. Whitsel, P. Miguel Quibrera, Richard L. Smith, Duanping Liao, Garnet L. Anderson, Ronald J. Prineas

**Affiliations:** 1 Epidemiologic Cardiology Research Center, Department of Epidemiology and Prevention, Division of Public Health Sciences, Wake Forest University School of Medicine, Winston-Salem, North Carolina, USA;; 2 Departments of Epidemiology; 3 Medicine and; 4 Statistics and Operations Research, University of North Carolina at Chapel Hill, Chapel Hill, North Carolina, USA;; 5 Department of Health Evaluation Sciences, Pennsylvania State University, Hershey, Pennsylvania, USA;; 6 Fred Hutchinson Cancer Research Center, Seattle, Washington, USA

**Keywords:** air pollution, cardiovascular disease, electrocardiography, myocardial ischemia, particulate matter

## Abstract

**Background:**

Ambient particulate matter (PM) air pollution is associated with coronary heart disease, but the pathways underlying the association remain to be elucidated.

**Methods:**

We studied the association between PM and ischemia among 57,908 Women’s Health Initiative clinical trial participants from 1999–2003. We used the Minnesota Code criteria to identify ST-segment and T-wave abnormalities, and estimated T amplitude (microvolt) from resting, standard 12-lead electrocardiogram (ECG). We used U.S. Environmental Protection Agency’s monitor data to estimate concentrations of PM < 2.5 μm (PM_2.5_) at geocoded participant addresses over 6 days before the ECGs (lag0 through lag5). We excluded 2,379 women with ECG QRS duration ≥ 120 msec.

**Results:**

Overall, 6% of the remaining 55,529 women (52–90 years of age; 83% non-Hispanic white) had ST abnormalities and 16% had T abnormalities. Lead-specific T amplitude was normally distributed (range of means from −14 to 349 μV). PM_2.5_ (mean ± SD) averaged over lag_0–2_ was 14 ± 7 μg/m^3^. In logistic and linear regression models adjusted for demographic, clinical, temporal, and climatic factors, a 10-μg/m^3^ increase in lag_0–2_ PM_2.5_ was associated with a 4% [95% confidence interval (CI), −3%, to 10%] increase in the odds of ST abnormality and a 5% (95% CI, 0% to 9%) increase in the odds of T abnormality. We observed corresponding decreases in T amplitude in all exam sites and leads except lead V1, reaching a minimum of −2 μV (95% CI, −5 to 0 μV) in lead V3.

**Conclusions:**

Short-term PM_2.5_ exposure is associated with ECG evidence of myocardial ischemia among postmenopausal women. The principal manifestations include subclinical but potentially arrhythmogenic ST–T abnormalities and decreases in T amplitude.

Studies of human populations show that ambient particulate matter (PM) air pollution is associated with coronary heart disease (CHD) morbidity and mortality. The consistent findings suggest direct associations between both short- and long-term exposure to PM and CHD incident [Brook et al. 2004; Dockery et al. 1993; Miller et al. 2007; Pope et al. 2006; U.S. Environmental Protection Agency (EPA) 2006]. However, biological mechanisms underlying the association between PM and CHD remain incompletely characterized in human populations despite calls for epidemiologic research designed to help elucidate them (Brook et al. 2004; Health Effects Institute 2002; National Research Council 2004).

Resting, supine, standard 12-lead electro-cardiography (ECG) has been recognized as a potentially valuable epidemiologic tool in this regard, yet to date much of its value remains unrealized. ECG findings characteristic of myocardial ischemia, for example, have not been adequately evaluated in this context. These measures include ST-segment depression and T-wave inversion on the ECG.

Because of their clinical value, these ECG findings have also attracted environmental, toxicologic, and epidemiologic interest. Indeed, they are thought to be relevant in studies focusing on arrhythmogenic effects of air pollution (Zareba et al. 2001) and risk factors for sudden death due to cardiac arrhythmia (Huikuri et al. 2001). The literature in this area has nevertheless focused on experimentation in animal models of coronary vasoconstriction (Campen et al. 2005; Godleski et al. 2000; Nikolov et al. 2007; Wellenius et al. 2003) or examination of typically small, single-city panels of CHD-burdened, Holter-monitored male participants during exercise (Gold et al. 2005; Henneberger et al. 2005; Lanki et al. 2006; Mills et al. 2007; Pekkanen et al. 2002; Yue et al. 2007). For this reason, epidemiologic evidence linking PM exposure to minor but prognostically important abnormalities of the ST segment and T wave in human populations at rest is quite limited.

We therefore designed the present study to investigate the association between acute exposures to ambient PM < 2.5 μm in diameter (PM_2.5_) and ECG measures of myocardial ischemia in a relatively large, geographically diverse, healthy female population enrolled in the Women’s Health Initiative (WHI) clinical trials.

## Methods

### Study design

We examined the association between PM_2.5_ and ischemia in the Environmental Epidemiology of Arrhythmogenesis in WHI (EEAWHI) study, an ancillary study of proarrhythmic mechanisms linking air pollution and CHD among 68,133 WHI clinical trial participants examined at 49 sites. The WHI clinical trial is a multicenter study of risk factors for prevention of common causes of mortality, morbidity, and impaired quality of life in U.S. postmenopausal women. Details of the study design, including recruitment procedures and selection and exclusion criteria, have been published elsewhere (WHI Study Group 1998). The study was approved by each study site’s institutional review board. All participants provided written informed consent.

### Study population

Of the 68,133 WHI clinical trial participants at baseline, we excluded 2,082 (3.1%) with foreign, U.S. military, U.S. protectorate, Hawaiian, Alaskan, or missing addresses; 417 (0.6%) with missing or poor-quality ECGs; 2,379 (3.5%) with ECG QRS duration ≥ 120 msec; and 7,726 (11%) without an ECG recording between 1999 and 2003, the initial 5-year period in which PM_2.5_ data were routinely collected by the U.S. EPA Air Quality System (AQS). The present study focuses on the initial high-quality, resting, standard 12-lead ECGs recorded during this period among the remaining 55,529 participants, all of whom had addresses in the contiguous United States.

### ECG methodology

Identical ECGs (MAC PC, Marquette Electronics, Inc., Milwaukee, WI) were used in all clinic sites, and resting, standard 12-lead ECGs were recorded for all participants using strictly standardized procedures. The chest electrodes were located in precise positions. All ECGs were processed in a central ECG laboratory (Epidemiological Cardiology Research Center, Wake Forest University, Winston-Salem, NC), where they were visually inspected for technical errors and inadequate quality. The ECGs were initially processed with the Dalhousie ECG program, and for the present study we reprocessed them with the 2001 version of the GE Marquette 12-SL program (GE Healthcare 2008; Prineas et al. 1982; Rautaharju et al. 1998). The Marquette measurement matrix contained several ECG measures of myocardial ischemia that we used as outcome variables in this study.

### ST-segment and T-wave amplitudes (microvolt)

We defined lead-specific depth (or height) of the ST segment (ST) 60 msec after the J point and T-wave amplitude (T) relative to the isoelectric line.

### ST, T, and ST–T abnormalities

We applied Minnesota Code (MC) criteria to the former interval-scale measures to identify ST abnormality (MC 4.1–4.4), T abnormality (MC 5.1–5.4), or any ST–T abnormality (MC 4.1–4.4 or MC 5.1–5.4) (Prineas et al. 1982).

### QRS/T angles (degrees)

The frontal plane QRS/T angle was defined as the absolute value of the difference between the frontal plane QRS and T axes (range, −89° to 270°). When > 180°, it was adjusted to the minimal angle as follows: 360° – frontal plane QRS/T angle (Zhang et al. 2007). We defined the spatial QRS/T angle as the angle between the mean QRS and T vectors, which we calculated from quasi-orthogonal X, Y, and Z leads reconstructed from the standard ECG leads via matrix transformation (Rautaharju et al. 2006b).

### Air pollutant concentrations, weather variables, and other participant characteristics

We obtained all ambient PM_2.5_ concentration data recorded at monitors operating in the contiguous United States during the study from the U.S. EPA AQS. The data included the longitude and latitude of each monitor. We used a semiautomated program built on ArcView GIS (version 8.3) software and its Geostatistical Analyst extension (ESRI, Inc. Redlands, CA) to produce kriging-estimated daily mean concentrations (and their SEs) at each geocoded participant and exam site address from the baseline examination through 2004 (Liao et al. 2006, 2007; Whitsel et al. 2006). The program relied on a spherical model to perform national-scale, lognormal ordinary kriging and the weighted least-squares method to estimate semivariograms, and ran and cross-validated each daily semivariogram. From the kriging-estimated, daily mean, location-specific PM_2.5_ concentrations, we then computed a matrix of 15 short-term pollutant exposures by averaging within overlapping 1-, 2-, and 3-day lag combinations inside a 6-day exposure window beginning 5 days before and ending on the examination date, that is, days 0–5. Associations between PM and ischemia were strongest on days 0–2 (lag_0–2_), the emphasis of the present report.

We obtained all meteorologic data recorded at relevant stations and times from the National Climatic Data Center (U.S. National Climatic Data Center 2007): ambient temperature (degrees centigrade), dew point (degrees centigrade), and barometric pressures (kilo pascal), as well as station longitudes, latitudes, and altitudes. Daily mean temperature, dew point, and pressure at each geocoded participant and exam site address from baseline to closeout were estimated by averaging daily means across all stations within 50 km, a distance over which their station-to-station correlations exceed 0.90 (Ito et al. 2001). Thereafter, we computed weather variables at lag_0–2_/lag_3–5_ as described above for PM_2.5_.

Self-reported education, medication use, health history, and a variety of other attributes were determined at each visit by standardized participant interview and examination by the WHI staff. Interim health events were identified via standardized medical record review and physician adjudication. Specifically, diabetes was defined by antidiabetic medication use or history; hypertension by antihypertensive medication use, systolic blood pressure (SBP) ≥ 140 mmHg, diastolic blood pressure (DBP) ≥ 90 mmHg, or history; body mass index (BMI) as the ratio of weight (kilograms) to height (square meters); hypercholesterolemia by antihyperlipidemic medication use or history; smoking as current, former, or never; chronic lung disease by history of asthma, emphysema, or lung cancer; CHD by antianginal medication use, history of angina, or myocardial infarction and medical record review/adjudication; revascularization by history of coronary artery angioplasty, stent, or bypass and medical record review/adjudication; and congestive heart failure by cardiac glycoside/diuretic use, history, and medical record review/adjudication.

### Statistical analysis methods

We inspected frequency distributions of all variables for outliers but did not identify influential values. Initially ignoring the influences of exam site, we explored the associations between ischemia and PM_2.5_ in unadjusted linear and logistic regression models. To control for residual confounding by exam season, day of week, time of day, health, and weather, we added temporal, demographic, clinical, and meteorologic characteristics to the models. We then conducted hierarchical analyses in two stages. In the first stage we used site-specific, adjusted regression models to estimate strength of the ischemia–PM_2.5_ associations. The models allowed these estimates to vary among sites. In the second stage we used random effects meta-regression of the site-specific, linear regression coefficients from the first stage to estimate overall strength of the ischemia–PM associations across sites. These weighted, iterative regression models allowed for an additive, between-study component of residual variance estimated via restricted maximum likelihood (Berkey et al. 1995). We repeated the second-stage analyses by placing uninformative priors on the second-stage parameters and Monte Carlo sampling from the conditional distribution of each unknown using standard Bayesian hierarchical regression algorithms (Robert and Casella 2004). We evaluated effect measure modification by including cross-product terms for the interaction between PM_2.5_ and CHD and its major risk factors at the first stage. Analyses based on logistic and linear regression models adjusting for exam site, and both the random-effects meta-analyses and Bayesian hierarchical regression analyses of the adjusted, site-specific regression coefficients produced similar results. We conducted all analyses using SAS version 9.1 (SAS Institute, Inc., Cary, NC) and MATLAB, version 7.0, software (MathWorks, Inc., Natick, MA). By convention, we report resulting estimates as odds ratios for ST, T, and ST–T abnormalities, or microvolt (μV) changes in ST-segment and T-wave amplitudes associated with 10 μg/m^3^ increases in PM_2.5_ concentrations.

## Results

Of the 55,529 female participants (age range, 52–90 years), 83% were non-Hispanic white, 52% hypertensive, and 20% hypercholesterolemia (Table 1). Somewhat smaller percentages were current smokers (6%) or had diabetes (8%), CHD (11%), or chronic lung disease (10%). Six percent of the study population had ST abnormalities, and 16% had T abnormalities. Lead-specific ST and T amplitudes were normally distributed (range of means = 4, 64 μV and −14, 349 μV, respectively). Mean ± SD spatial and frontal plane QRS/T angles were 60° ± 28° and 29° ± 27°, with heart rate 66 ± 10 beats/min (Table 2). Mean PM_2.5_ averaged over the first 3 days and second 3 days preceding the ECG (lag_0–2_ and lag_3–5_) were equivalent, as were mean temperature, dew point, and barometric pressure (Table 3).

**Figure 1:**
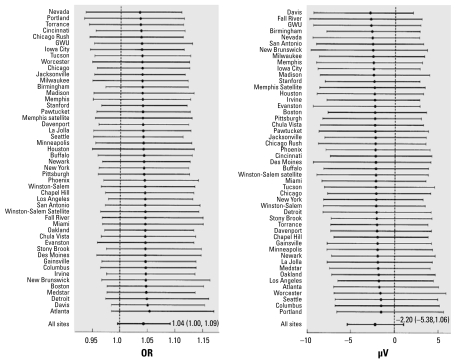
Multivariable-adjusted ORs for ST–T abnormality and changes in T amplitude (μV) in lead V3 (95% CI) per 10-μg/m^3^ increase in lag_0–2_ PM_2.5_, by exam site. We based estimates on Bayesian hierarchical regression models adjusted for demographic, clinical, and weather variables: age; race/ethnicity; education; BMI; current smoking status; history of CHD, diabetes, hypertension, SBP, chronic lung disease, or hypercholesterolemia; season; day of week; time of day; temperature; dew point; and pressure.

In logistic and linear regression models adjusted for demographic, clinical, temporal, and climatic factors, 10 μg/m^3^ increases in lag_0–2_ PM_2.5_ were associated with 4–5% increases in odds ratios (ORs) for ST, T, and ST–T abnormalities, small decreases in lead-specific ST and T amplitudes, and slight increases in the QRS/T angles and heart rate (Table 4). We observed decreases in T amplitude in all leads except V1, reaching a minimum of −2.3 μV [95% confidence interval (CI), −5.2 to 0.5 μV] in lead V3 even after controlling for its reported dependence on heart rate (Couderc et al. 2007). These observations were consistent across exam sites (Figure 1). The observed magnitude and direction of effects associated with lag_3–5_ PM_2.5_ were often weaker and less consistent [Table 4 and Supplemental Material, Tables 1 and 2 (available online at http://www.ehponline.org/members/2009/0800046/suppl.pdf)]. Findings based on unadjusted models and those controlling for randomization status as well as additional participant- and contextual- level measures of education, occupation, income, and housing were comparable.

Effects of lag_0–2_ PM_2.5_ were slightly stronger among users versus nonusers of beta-blockers: 10% (1%, 20%) versus 3% (−1%, 8%) increases in the odds of T abnormality (*p* = 0.098) and −8 (−15, −1) versus −2 (−4, 1) μV decreases in T amplitude in lead V3 (*p* = 0.076), but in general, there was little suggestion of effect measure modification by CHD or its major risk factors.

## Discussion

Judging from the U.S. EPA’s *Provisional Assessment of Recent Studies on Health Effects of Particulate Matter Exposure* (National Center for Environmental Assessment 2006), interest in subclinical, atherosclerotic manifestations of long-term ambient PM_2.5_ exposure has dramatically increased. Indeed, the July 2006 assessment included only one such study (of coronary artery calcium), yet in the interim, several others have also examined associations between PM_2.5_ exposures sustained over months to years and noninvasive measures of atherosclerosis, including carotid intimal-medial thickness, abdominal aortic calcification, and ankle-brachial index (Allen et al. 2009; Diez Roux et al. 2008; Hoffmann et al. 2007; Kunzli et al. 2005). The epidemiologic associations observed in these studies are not entirely consistent with one another or collectively as strong as those that may have been anticipated on the basis of recently reported associations between chronic PM exposure and cardiovascular disease (e.g., Miller et al. 2007), and the role of atherosclerotic mechanisms in the association remains uncertain.

We examined the role of acute, ischemic mechanisms in the association between PM_2.5_ and CHD among WHI clinical trial participants with this uncertainty in mind, using ECG—a simple, noninvasive, and underused research tool. In this epidemiologic context, however, we did not limit the examination to characteristics of clinically manifest ischemia (e.g., ≥ 0.1 mV of downsloping ST segment depression and/or deep, symmetrical, pre-cordial T-wave inversion). We opted instead to include minor abnormalities of ventricular repolarization (ST–T abnormalities, ST and T amplitudes) and depolarization (QRS/T angles) to increase sensitivity of the ECG for subclinical forms of myocardial ischemia. Using this tool, we found that a 10 μg/m^3^ increment in lag_0–2_ PM_2.5_ is associated with 4–5% increased odds of minor ST, T, and ST–T abnormalities, small decreases in ST and T amplitudes across most leads and exam sites, and corresponding increases in QRS/T angles.

Previous findings from the WHI clinical trials cohort include significant prospective relationships between isolated ST–T abnormalities, lead-specific ST and T amplitudes, and wide QRS/T angle at baseline, and CHD mortality, incident CHD, and congestive heart failure (Rautaharju et al. 2006b). Similar findings also have been described in population-based settings, such as the Atherosclerosis Risk in Communities study (Zhang et al. 2007), Cardiovascular Health Study (Rautaharju et al. 2006a), and Chicago Western Electric Study (Daviglus et al. 1999), among others (Engel et al. 2004; Larsen et al. 2002; Okin et al. 2005, 2007; Schouten et al. 1992). However, the clinical utility of these ECG measures remains limited.

Considering the consistent associations between PM_2.5_ and the ECG measures of myocardial ischemia observed in this context and the established, prospective association of these measures with CHD, the findings from the present study provide additional support for the hypothesis that subclinical myocardial ischemia has a pathophysiologic role in the adverse effects of acute exposure to PM_2.5_ on the heart, including predisposition to ventricular arrhythmogenesis and sudden cardiac death (Rubart and Zipes 2005). This is the first study to examine such an association in a large population of postmenopausal women. Indeed, prior studies have focused on either animal models of coronary vasoconstriction (Campen et al. 2005; Godleski et al. 2000; Nikolov et al. 2007; Wellenius et al. 2003) or typically small, single-city panels of CHD-burdened, Holter-monitored male participants during exercise (Gold et al. 2005; Henneberger et al. 2005; Lanki et al. 2006; Mills et al. 2007; Pekkanen et al. 2002; Yue et al. 2007) (Table 5). The findings described herein are nonetheless consistent with and thereby extend the findings previously described in these populations. They do not imply, however, that ischemia is the sole process by which PM_2.5_ exerts its adverse effects on the heart. Autonomic, thrombotic, endothelial, inflammatory, oxidative, and other mechanisms of disease have all been implicated in cardiovascular effects of PM (Chuang et al. 2007; Mills et al. 2007).

This study has several limitations that may affect interpretation of its findings. First, it is an ancillary study of participants in the WHI clinical trials. Inference is therefore limited to postmenopausal women 52–90 years of age living in the contiguous 48 United States between 1999 and 2003, the initial 5-year period during which PM_2.5_ data were routinely collected by the U.S. EPA AQS. Because women in the WHI clinical trials were randomized to estrogen with or without progestin treatment, calcium/vitamin D supplementation, and/or dietary modification, these exposures could have affected the ECG measures examined in this setting. Because ambient PM_2.5_ concentrations also decreased 11% in the United States between 2000 and 2007 (U.S. EPA 2008a), observed associations between PM and ischemia could be weaker if this study were repeated today. Findings were nevertheless robust to adjustment for randomization status. Moreover, seasonally weighted annual average PM_2.5_ concentrations in the United States still exceed the 15 μg/m^3^ national ambient air quality standard at approximately 10% of trend sites (U.S. EPA 2008b).

Second, we did not measure PM_2.5_ in the personal breathing space of participants. Instead, it was spatially interpolated at each participant’s geocoded address, raising questions about its validity (Szpiro et al. 2007). Third, the associations described herein also may be subject to residual socioeconomic confounding because we defined diabetes and hypercholesterolemia by self-reported history of the disease and/or use of antihyperlipidemic or antidiabetic medications rather than fasting glucose or lipid concentrations, which were only available for 6% site- and race-stratified minority participant oversample.

In the face of these additional limitations, in this study we took several precautions. We carefully established the validity of the geo-codes relative to criterion standards (Whitsel et al. 2006) and the spatial interpolations using standard cross-validation statistics: the prediction error (PE = predicted minus measured pollutant concentration at each monitor site), standardized prediction error (SPE = PE divided by its estimated standard error), root mean square standardized error (RMSS = standard deviation of SPE across sites), root mean square prediction error (RMS = empirical standard error based on the mean square of the predictions), and mathematically calculated standard error (SE). Observed values of PE and SPE near 0 and RMSS near 1, and similarity of RMS and SE provide evidence of model validity (Liao et al. 2006, 2007). In addition, the study documented modest effects of geocoding and kriging error on PM–CHD associations (Crooks et al. 2008; Whitsel et al. 2006). Finally, the study demonstrated high reliability of treated diabetes and hypercholesterolemia (Langer et al. 2003), and we adjusted effect sizes for additional participant- and contextual-level measures of socioeconomic status, noting only small changes as a result of adjustment.

## Conclusion

We conclude that short-term exposure to PM_2.5_ is associated with ECG evidence of myocardial ischemia among postmenopausal women. The principal manifestations include ST-segment and T-wave abnormalities, decreases in ST segment and T amplitude, and increases in QRS/T angles. These findings suggest that the adverse effects of PM_2.5_ on CHD risk among postmenopausal women may be related to its subclinical, ischemic effects on the myocardium.

## Figures and Tables

**Table 1:** The demographic and clinical characteristics of the study population on the date of ECG examination (*n* = 55,529).

Characteristic	Mean ± SD or percent
Age in years on ECG exam date	67 ± 7
Race	
White	83
Black	10
Other	7
Education < college	23
BMI (kg/m^2^)	29 ± 6
SBP (mmHg)	126 ± 17
DBP (mmHg)	73 ± 9
Hypertension	52
Hypercholesterolemia	20
Diabetes	8
Current smoking	6
CHD^a^	11
Chronic lung disease	10
Beta-blocker use	13
Exam season	
Spring	27
Summer	26
Fall	25
Winter	22
Exam day	
Monday–Thursday	86
Friday	12
Saturday–Sunday	2
Exam time	
Morning	64
Afternoon	36

a CHD includes congestive heart failure, coronary revascularization, or myocardial infarction by Minnesota Code or Novacode.

**Table 2:** The ECG parameters of the study population on the date of ECG examination (*n* = 55,529).

ECG parameter	Mean ± SD or percent
Minnesota codes	
MC 4^a^	6
MC 5^b^	16
MC 4 or MC 5^c^	18
T-wave amplitude (μV)	
Lead I	202 ± 105
Lead II	241 ± 105
Lead aVL	86 ± 93
Lead V1	−14 ± 116
Lead V2	313 ± 197
Lead V3	349 ± 203
Lead V4	324 ± 193
Lead V5	284 ± 167
Lead V6	221 ± 126
ST-segment amplitude (μV)	
Lead I	13 ± 21
Lead II	19 ± 25
Lead aVL	4 ± 17
Lead V1	27 ± 27
Lead V2	64 ± 46
Lead V3	47 ± 41
Lead V4	29 ± 35
Lead V5	18 ± 29
Lead V6	11 ± 23
QRS/T angles and heart rate	
Heart rate (beats/min)	66 ± 10
QRS/T angle–spatial (°)	60 ± 28
QRS/T angle–frontal plane (°)	29 ± 27

a Any ST abnormality (MC 4.1–4.4).

b Any T abnormality (MC 5.1–5.4).

c Any ST–T abnormality (MC 4.1–4.4 or MC 5.1–5.4).

**Table 3:** PM concentrations and weather variables for the study population on the date of ECG examination.

Characteristic	Mean ± SD
PM_2.5_^a^	
Lag_0_	14.1 ± 8
Lag_1_	13.8 ± 8
Lag_2_	13.8 ± 8
Lag_3_	13.8 ± 8
Lag_4_	13.9 ± 8
Lag_5_	14.1 ± 8
Lag_0–2_^b^	13.9 ± 7
Weather	
Temperature lag_0–2_^b^	13.9 ± 9
Dew point lag_0–2_^b^	7.6 ± 9
Barometric pressure lag_0–2_^b^	102 ± 1

a Concentrations at geocoded participant addresses on day of exam (lag_0_) and preceding 5 days (lag_1–5_).

b The mean ± SD for PM_2.5_ and weather variables were the same at lag_3–5_.

**Table 4:** Multivariable-adjusted^a^ ORs and changes (95% CI) per 10-μg/m^3^ increase in PM_2.5_.

ECG parameter	Lag_0–2_ (95% CI)	Lag_3–5_ (95% CI)
Minnesota Codes (OR)		

MC 4^b^	1.04 (0.97 to 1.10)	1.04 (0.98 to 1.11)
MC 5^c^	1.05 (1.00 to 1.09)	1.04 (1.00 to 1.08)
MC 4 or MC 5^d^	1.04 (1.00 to 1.09)	1.03 (0.99 to 1.07)

ST-segment amplitude (μV, change)		

Lead I	−0.07 (−0.36 to 0.21)	0.18 (−0.10 to 0.46)
Lead II	−0.12 (−0.47 to 0.23)	0.16 (−0.18 to 0.50)
Lead aVL	−0.01 (−0.25 to 0.23)	0.11 (−0.12 to 0.34)
Lead V1	−0.02 (−0.39 to 0.35)	−0.22 (−0.58 to 0.14)
Lead V2	0.07 (−0.57 to 0.70)	−0.01 (−0.61 to 0.62)
Lead V3	−0.11 (−0.68 to 0.47)	−0.02 (−0.58 to 0.54)
Lead V4	−0.03 (−0.51 to 0.45)	0.24 (−0.23 to 0.71)
Lead V5	−0.01 (−0.41 to 0.39)	0.35 (−0.04 to 0.74)
Lead V6	0.02 (−0.30 to 0.33)	0.35 ( 0.04 to 0.65)

T-wave amplitude (μV, change)		

Lead I	−1.60 (−3.07 to −0.13)	−0.31 (−1.73 to 1.11)
Lead II	−0.54 (−1.99 to 0.92)	0.71 (−0.70 to 2.13)
Lead aVL	−1.21 (−2.50 to 0.10)	−0.55 (−1.81 to 0.71)
Lead V1	1.45 (−0.16 to 3.06)	0.03 (−1.53 to 1.59)
Lead V2	−0.18 (−2.96 to 2.60)	0.57 (−2.12 to 3.27)
Lead V3	−2.33 (−5.15 to 0.49)	−0.13 (−2.87 to 2.60)
Lead V4	−2.03 (−4.69 to 0.63)	0.64 (−1.94 to 3.22)
Lead V5	−1.92 (−4.22 to 0.38)	0.55 (−1.69 to 2.78)
Lead V6	−0.63 (−2.36 to 1.10)	0.82 (−0.86 to 2.49)

QRS/T angles and heart rate (change)		

QRS/T angle–spatial (°)	0.19 (−0.21 to 0.59)	−0.20 (−0.59 to 0.19)
QRS/T angle–frontal plane (°)	0.13 (−0.24 to 0.50)	0.35 (−0.01 to 0.71)
Heart rate (beats/min)	0.16 ( 0.02 to 0.30)	0.04 (−0.10 to 0.18)

a Estimates based on logistic and linear regression models adjusted for demographic, clinical, and weather variables: age; race/ethnicity; education; exam site; BMI; current smoking status; history of CHD, diabetes, hypertension, SBP, chronic lung disease, or hypercholesterolemia; day of week; time of day; temperature; dew point; pressure and season;

b Any ST abnormality (MC 4.1–4.4).

c Any T abnormality (MC 5.1–5.4).

d Any ST–T abnormality (MC 4.1–4.4 or MC 5.1–5.4).

**Table 5:** Human studies of association of PM and ischemia.

Reference	No.	Location	Mean age (years)	Female (%)	Outcomes	Detection method
Pekkanen et al. 2002	45	Helsinki, Finland	68	47	STD	E Holter
Henneberger et al. 2005	56	Efurt, Germany	66	0	TWA	R Holter
Gold et al. 2005	24	Boston, MA, USA	73	75	STD	E Holter
Lanki et al. 2006	45	Helsinki, Finland	68	47	STD	E Holter
Yue et al. 2007	56	Efurt, Germany	66	0	TWA	R Holter
Mills et al. 2007	20	Southampton, UK	60	0	STD	E Holter
Zhang et al. 2008	55,529	49 exam sites^a^	67	100	STT, STA, TWA	Resting ECG

Abbreviations: E, exercise; R, rest; STA, ST-segment amplitude; STD, ST-segment depression; STT, ST-segment and T-wave abnormalities; TWA, T-wave amplitude. ECG involved resting, supine, standard 12-lead ECG.

a In the contiguous United States.
